# Charge for peer reviewing

**DOI:** 10.1590/S1679-45082014CE3287

**Published:** 2014

**Authors:** Lucas Vieira dos Santos

**Affiliations:** Instituto de Ensino e Pesquisa São Lucas, São Paulo, SP, Brazil.

“There’s no such thing as a free lunch” is an adage described over one century ago, which became popular by Professor Milton Friedman, in 1975. It came to my mind when reading the interesting editorial by Pasternak and Glina.^(1,2)^ Working on both sides, as an author of journals and a reviewer, I am aware of the importance of a good review. It is powerful to enhance the description of results in an article, in addition to highlighting its strengths and limitations, which are ultimately translated in improving articles of a certain journal. Many times I have felt grateful for the unknown hero – the reviewer – for the contribution made to a work I had submitted.

It can be stated that, when the services of a reviewer are “bought”, what he/she finds more difficult is to provide their time (and not their expertise), which limits the possibility of accepting the task of review. Therefore, it is very fair to pay for such relevant work. However, each journal should decide how to do it according to its reality.

Lucas Vieira dos Santos *Instituto de Ensino e Pesquisa São Lucas*, São Paulo, SP, Brazil.
