# Supplementary material for: Charge for peer reviewing
Source: Einstein (Sao Paulo). 2014 Oct-Dec;12(4):534. doi: 10.1590/S1679-45082014CE3287 (PMC4879929; doi:10.1590/S1679-45082014CE3287)
Supplement: Translation

# Translation: Pagamento para revisão em pares

**Authors:** Lucas Vieira dos Santos

**Affiliations:** Instituto de Ensino e Pesquisa São Lucas, São Paulo, SP, Brasil.

“Não existe almoço grátis” é um adágio de mais de um século, popularizado pelo Professor Milton Friedman, em 1975, e que me veio à mente ao ler o interessante editorial de Pasternak e Glina.^(1,2)^ Estando dos dois lados, como autor de periódico e como revisor, sei da importância de uma boa revisão. Ela tem grande poder em melhorar a exibição dos resultados de um artigo, bem como de incrementar os pontos fortes e limitações, o que se traduz, em última instância, em melhoria dos artigos daquele periódico. Em inúmeras vezes eu senti gratidão por aquele herói desconhecido, o revisor, por sua contribuição com o trabalho que eu havia submetido.

Quando se “compra” o serviço de um revisor, esteja certo de que o que ele tem mais dificuldade em oferecer é seu tempo, pois é o tempo livre (e não a *expertise*) que limita a possibilidade de aceite de revisão. Nada mais justo, portanto, do que haver remuneração para esse importante trabalho. A forma que isso deve ocorrer, entretanto, deve ser individualizada para a realidade de cada periódico.

Lucas Vieira dos Santos *Instituto de Ensino e Pesquisa São Lucas*, São Paulo, SP, Brazil.
